# Gingival Enlargement Induced by Felodipine Resolves with a Conventional Periodontal Treatment and Drug Modification

**DOI:** 10.1155/2016/1095927

**Published:** 2016-02-29

**Authors:** Nabil Khzam, David Bailey, Helen S. Yie, Mahmoud M. Bakr

**Affiliations:** ^1^DB Dental, Corner Tydeman & Pensioner Guard Roads, Perth, WA 6159, Australia; ^2^Irwin Dental Centre, Irwin Barracks, Perth, WA 6010, Australia; ^3^General Dental Practice, School of Dentistry and Oral Health, Griffith University, Gold Coast, QLD 4222, Australia

## Abstract

We present a case of a 47-year-old male who suffered from GE around his lower anterior teeth as soon as he started treatment with Felodipine 400 mg. We show that oral hygiene measures, antibiotics, and conventional periodontal treatment (scaling and root planing SRP) were all not sufficient to resolve the drug induced GE, which will persist and/or recur provided that systemic effect of the offending medication is still present. The condition immediately resolved after switching to a different medication. The mechanism of GE is complex and not fully understood yet. It is mainly due to overexpression of a number of growth factors due to high concentrations of calcium ions (Ca^2+^). This affects fibroblasts proliferation and DNA synthesis and leads to a heavy chronic inflammatory cell infiltrate. Our case was managed according to the suggested protocols in previous case studies. The unique features in our case were the immediate onset of the adverse effect after starting the medication and the absence of any underlying medical condition apart from high blood pressure. Improving the oral hygiene together with SRP and cessation of the medication resolves drug induced GE.

## 1. Introduction

Different medications can cause a number of adverse reactions in the oral cavity including but not limited to oral ulceration, xerostomia, lichenoid reactions, oral pigmentations, burning mouth syndrome, tooth discoloration, and gingival hyperplasia [1]. Gingival enlargement (GE), also known as gingival overgrowth or hyperplasia, has a multifactorial aetiology including inflammation, neoplastic conditions, systemic disorders, and medications [2]. Drugs associated with GE can be grouped into anticonvulsant drugs (phenytoin) [3–5], potent immunosuppressants (cyclosporin) [6–8], and specific antihypertensive drugs (calcium channel blockers) [9–16].

The aetiology for GE is not fully understood. However, there has been a different correlation to different inflammatory and noninflammatory pathways. Individual's reaction or sensitivity towards a metabolic pathway could be a contributing factor as well [17]. Other nutritional and/or environmental factors may also play a role [18]. Untreated hyperplastic gingival tissue may lead to aesthetic, functional, and periodontal drawbacks and difficulties that affect the patient's well-being and may lead to an increased treatment cost on the long run [19].

Different studies aimed to investigate the possible factors contributing to drug induced GE. A study performed on patients receiving Phenytoin showed that the expression of some growth factors including Transforming Growth Factor (TGF-*β*1) and Platelet Derived Growth Factor (PDGF-BB) was significantly higher in GE areas when compared to nonenlarged gingival sites in the same patient and control patients not receiving Phenytoin [20]. Combinations of different drugs were also investigated and it was reported that these combinations increase the incidence and severity of GE [21]. A notorious combination is Cyclosporin and Nifedipine as the later could counteract the former's side effects of nephrotoxicity and hypertension [22]. It was proven that the above-mentioned combination increases the prevalence and intensity of GE when compared to using Cyclosporin with Amlodipine or Cyclosporin alone [23]. Furthermore, prevalence of Amlodipine induced GE is as low as 1.3% [24].

## 2. Case Presentation

**Figure 1:**
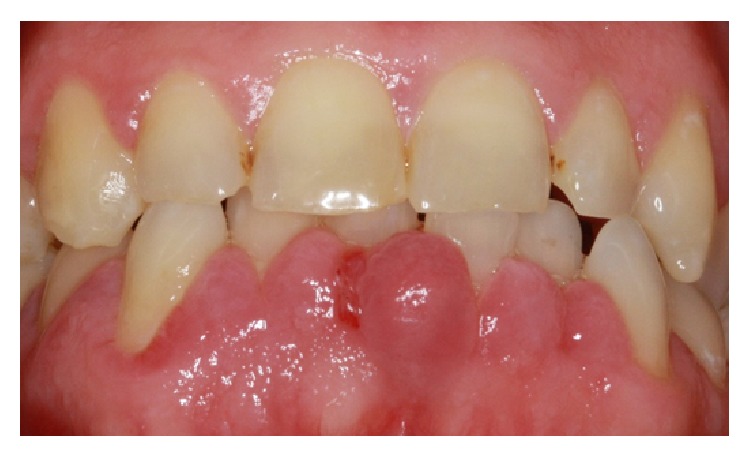
A photo showing the initial presentation of the GE associated with all lower anterior teeth.

A 47-year-old male initially presented to a General Dentist for a regular dental check-up, where his enlarged gingiva was noted. On examination, there was gingival hyperplasia and pocketing of 5 mm (Figure 1). The patient was in pain and reported that the swelling started few days after a new antihypertensive medication (Felodipine) was prescribed. This was the first time ever the patient has had this kind of reaction to any medication as well as being the first time the patient experiences any gingival swellings. There was no pain associated with the swelling initially, but due to minimal trauma the pain started; small amount of bleeding was associated with the trauma.

A review of the patient's medical history revealed nothing significant other than high blood pressure (160/95) and family history of hypertension (father). A review of patient's medications showed that two months before presenting to the General Dentist, the patient was placed on Telmisartan 80 mg (Micardis) 28 tablets 1 tab daily and Felodipine (Plendil) 10 mg 30 tablets take 1 tab daily. A review of oral hygiene measures revealed the use of manual toothbrush twice daily but no flossing. Patient‘s oral hygiene was poor. Patient is a regular attender to dental appointment once a year for check-ups and cleans.

The General Dentist performed a deep scale and clean, removed some of the gingival tissue that is constantly being traumatized due to the swelling and prescribed Metronidazole 400 mg tid for a week, Panadeine Forte (Paracetamol 500 mg + Codeine Phosphate 30 mg), Ibuprofen 400 mg, and Savacol mouthwash (2 mg/mL Chlorhexidine Gluconate). There was no improvement in the gingival swelling despite the above treatment. However, the pain decreased.

The patient was referred to a specialist periodontist. A full comprehensive examination was done and revealed a periodontal diagnosis of mild to moderate generalized Chronic Periodontitis modified by poor oral hygiene and plaque induced GE affecting lower anterior teeth. The proposed treatment plan was patient education, oral hygiene instructions, SRP, surgical removal of the GE, the removed tissue to be submitted for a biopsy, reevaluation of periodontal tissue conditions in 3 months after completion of the treatment and supportive periodontal treatment. Upper and lower Jaw debridement under local anaesthesia and an excisional biopsy including all labial GE around the lower anterior teeth were performed by the specialist periodontist. Communications to the patient's general practitioner regards the potential side effects of the antihypertensive medication and the possible correlations with the GE. The patient's general practitioner changed the antihypertensive medications to Coversyl (Perindopril) 30 tablets 10 mg 1 tab daily and Felodipine (Plendil) 10 mg 30 tablets take 1 tab daily.

**Figure 2:**
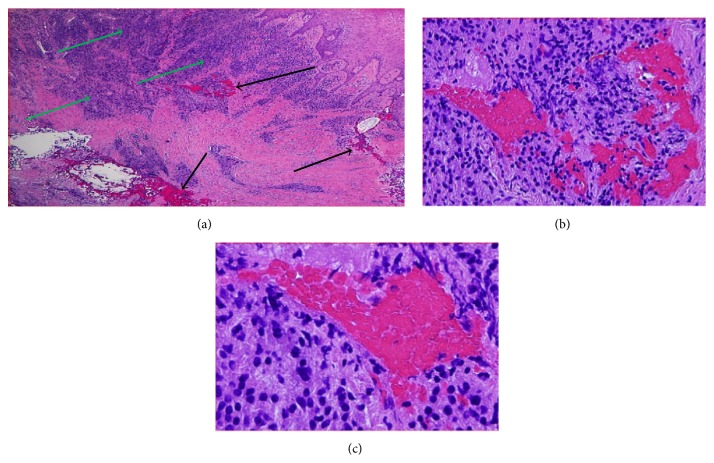
A photo showing the histopathological picture of the GE. (a) Showing heavy inflammatory cell infiltrate (green arrows) and osseous metaplasia (black arrows), H&E x2. (b) Showing a higher magnification of some areas of osseous metaplasia, H&E x20. (c) Showing an area of osseous metaplasia surrounded by chronic inflammatory cells, H&E x40.

**Figure 3:**
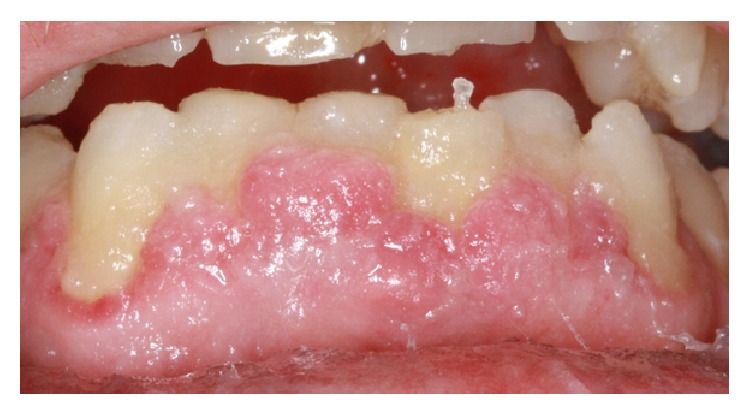
A photo showing the persistence of GE after the initial periodontal treatment. The inflammation decreased slightly. Oral hygiene improved after the initial treatment.

Biopsy results showed the diagnosis was Fibrous Epulis with osseous metaplasia (Figure 2). In the first review appointment, the labial GE was still present. However, the degree of inflammation decreased (Figure 3). The overall oral hygiene of the patient was reasonable. Patient was scheduled for a second review in three months. Concerns were raised that Felodipine might be the cause of GE. Another communication to the patient's general practitioner resulted in changing his medication to Telmisartan 80 mg (Micardis) 28 tablets 1 tab daily and Moxonidine 400 mg (Physiotens) 30 tablets 1 tab daily.

**Figure 4:**
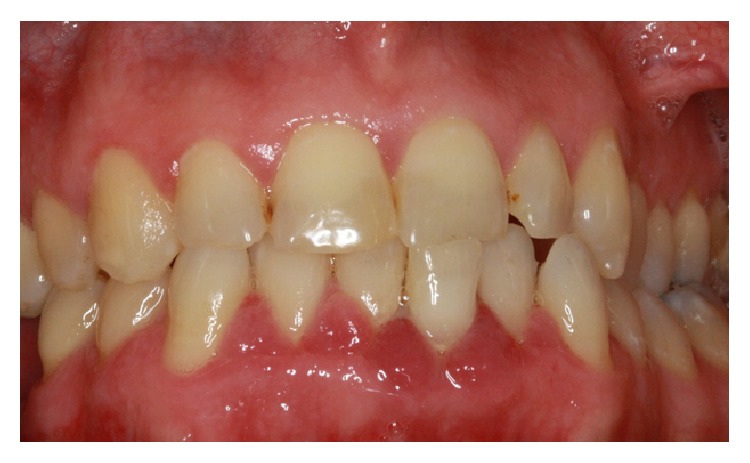
Showing disappearance of the GE and improvement of the overall periodontal condition after cessation of Felodipine together with strict oral hygiene measures.

**Figure 5:**
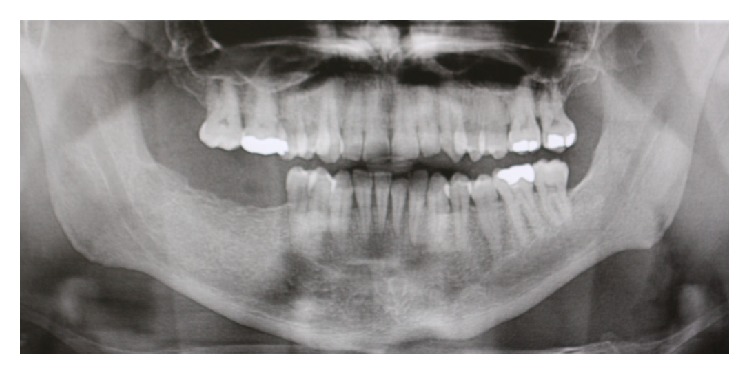
An orthopantograph (OPG) showing mild to moderate bone loss around lower anterior teeth, which compromises the long term prognosis of these teeth and mandates strict oral hygiene measures and careful and regular monitoring.

The specialist periodontist contacted the patient, who reported disappearance of the GE immediately after cessation of Felodipine. In the second review appointment, there was a significant reduction in GE (Figure 4). A panoramic X-ray (OPG) was taken to be used as a base line to monitor the bone levels after regression of GE. The OPG showed mild to moderate horizontal bone around the lower anterior teeth (Figure 5). The importance of attending regular recall appointments in order to maintain the periodontal health of all teeth and specifically the lower anterior teeth was highly stressed.

## 3. Discussion

The response of gingival fibroblasts to calcium channel blockers has been investigated and mainly attributed to several mechanisms including high intracellular free Ca^2+^ concentration which results in cellular responses that affects growth factors, cell cycle regulators, cell proliferation, DNA, and collagen synthesis as well as intracellular cell talk [25]. Several therapeutic treatments were investigated in order to deplete the intracellular storage of Ca^2+^ including Tenidap and showed a degree of success on human cultured gingival fibroblasts [25].

Felodipine is an extended-release (ER) formula used to avoid the need for multiple daily doses and minimize side effects. When comparing the adverse effects Felodipine against those of other calcium channel blockers such as Nifedipine, a greater number of patients suffer from ankle oedema, nausea, and headache after receiving treatment with the former medication for mild to moderate hypertension [26]. This difference may be due to the different pharmacokinetic profiles of these two dihydropyridines; that is, with Felodipine ER the acute vasodilatory side effects are thought to be related to a rapid onset of action, high peak plasma concentrations, and large peak-trough concentration ratios. Such kinetic properties cause reflex sympathetic activation leading to the previously mentioned side effects [27]. We believe that the GE could be attributed to the same above-mentioned pharmacological properties and mechanism.

Our histological findings were identical to another case report related to GE induced by Nifedipine. In both cases there was a heavy infiltration with chronic inflammatory cells and fibroblast proliferation [28, 29] which indicates a common mechanism is shared between both types of antihypertensive drugs regardless of their class and/or longevity of action. In addition to the above, there were osseous metaplasia and separate islands of bone seen in the deeper layers of gingival tissues. This can be explained by the presence of an underlying periodontal problem that adds to the adverse effects of the medication. Therefore, there is a high possibility that the lesion might reoccur if the plaque induced causative factor is not removed through conventional periodontal treatment.

In a previous case report of GE induced by Felodipine, it was shown that stopping the medication led to complete resolution of the gingival condition without any clinical intervention such as SRP or home care and oral hygiene measures [2]. It should be noted that in the above-mentioned case report the patient had an uncontrolled type II diabetes mellitus. In our case report, the patient was medically fit (apart from hypertension) and had no underlying medical problems that could have contributed to the GE other than the antihypertensive medication. In both cases no surgical interventions were needed to eliminate the GE permanently.

GE induced by calcium channel blockers does not require treatment with antibiotics as it is not a result of bacterial infection. This is in contrast with cases related to Cyclosporine-A where adjunctive treatment with Roxithromycin together with SRP decreased levels of Transforming Growth Factor (TGF-*β*1) in gingival crevicular fluid, which improved the state of gingival tissue in immunocompromised patients [30]. We believe that the underlying suppressed immune system of patients on Cyclosporine-A justifies the need for supportive antibiotic therapy to enhance treatment outcomes.

With regard to the long term response to treatment in cases of GE, a recent study monitored patients with calcium channel blockers induced GE for 11 years and showed that 47.2% of patients suffered from recurrence of the GE during supportive periodontal therapy. In addition to that the long term tooth loss was higher in patients receiving calcium channel blockers [31]. Finally, replacement or withdrawal of the calcium channel blockers resulted in improvement of the GE; however, the condition did not heal completely, which indicates that there is an element of permanent damage that occurs after the use of these medications. The question of whether the oral hygiene status affects the long term prognosis is still debatable. Therefore, continuous follow-up is essential for our case in order to shed some light on the factors that influence a successful treatment outcome.

The key factors in our case management were drug substitution together with plaque control. This is in agreement with another case related to Amlodipine in a geriatric patient and the same treatment protocol was followed successfully [32]. Our biopsy results showed increased inflammatory cells infiltrate which is a very common well-documented feature of GE. Some authors even believe that the term gingival hyperplasia is not accurate as the GE does not result from an overproduction of cells. Instead it is a consequence of increased extracellular fluid due to chronic inflammatory cell infiltrate especially B-lymphocytes [33].

Recently, a universal hypothesis related to the mechanism of all drug categories that induce GE was suggested. Decreased cation influx of folic acid active transport within gingival fibroblasts leads to decreased cellular folate uptake, which in turn leads to changes in matrix metalloproteinases metabolism and the failure to activate collagenase. Decreased availability of activated collagenase results in decreased degradation of accumulated connective tissue which leads to GE [34]. Despite the fact that a universal theory for pathogenesis of drug induced GE could be accepted, the presentation of cases shows great variations. A classic case usually involves a high dose of the medication that has been used for at least 3–6 months. In our case the GE started immediately after using Felodipine. In another case, GE was evident even with a small dose (5 mg) of Amlodipine [35].

Cessation of the offending drug and its replacement with a different one are a crucial step in management of drug induced GE. Even switching to another medication of the same therapeutic class can lead to a remarkable improvement [36]. Similarly switching Cyclosporin A to Tacrolimus in organ transplant patients resulted in control of GE [37]. In our case, switching the medication from Felodipine to Moxonidine (imidazoline_1_ agonist) resulted in an immediate regression of the GE. Moxonidine exerts its blood pressure-lowering effect through stimulation of imidazoline type 1 (I_1_) receptors in the cardiovascular regulatory centres of the medulla oblongata [38]. Up to the authors' knowledge, cases of GE associated with Moxonidine have not been reported. However, dry mouth (xerostomia) is a known side effect of this medication [39]. Therefore, this should be taken into consideration while planning for our patient's ongoing periodontal care. Regular follow-up appointments and maintaining periodontal health are important for the long term successful management of drug induced GE cases [40].

Furthermore, Moxonidine has an inhibitory effect on the sympathetic nervous system. Therefore, it produces an antihypertensive effect that is equivalent or superior to other classes of antihypertensive medications (including Felodipine) that act centrally on the nervous. In addition to the above, Moxonidine showed to have a better tolerance profile with less incidence of the most common side effects [41]. Of particular interest to our case, Moxonidine was reported to have an anti-inflammatory effect that is secondary to the decrease in the sympathetic system activity [42]. This potential anti-inflammatory effect would have a positive effect in counteracting any preexisting GE conditions and would explain the absence of documented cases of GE in conjunction with Moxonidine treatment.

Our case is unique as there was an immediate response to the change in medication within days. This is in contrast to other GE cases where regression of GE takes longer periods up to six months [19, 33]. The osseous metaplasia in the histopathological picture in our case was not previously documented. It is attributed to the presence of an existing periodontal condition that affects bone turnover. The bony islands are associated with the inflammatory GE condition and are expected to disappear after regression of GE and control of the periodontal condition. We endeavour to follow-up on this case and report any cases of recurrence of GE.
